# Australian General Practitioners’ perspectives, experiences and use of non-drug interventions in primary care: a qualitative study

**DOI:** 10.1136/fmch-2025-003741

**Published:** 2026-01-06

**Authors:** Alexandra R. Davidson, Hannah Greenwood, Isabella Maugeri, Caroline Katherine Dowsett, Loai Albarqouni

**Affiliations:** 1 Institute for Evidence-Based Healthcare, Bond University, Robina, Queensland, Australia; 2 Bond University Faculty of Health Sciences and Medicine, Gold Coast, Queensland, Australia

**Keywords:** General Practice, Primary Health Care, Physicians, Primary Care, Qualitative Research, Health Services Research

## Abstract

**Objective:**

Non-drug interventions (NDIs) are underused in primary care, despite established effectiveness, safety, cost–benefit and guidelines. Existing research exploring barriers and enablers to NDI use primarily focuses on patients’ perspectives, leaving general practitioners’ (GPs’) perspectives underexplored, despite their critical role in NDI delivery. The objective of this study is to explore Australian GPs’ experiences and perspectives on the use of NDIs in primary care.

**Design:**

An interview study informed by realist methodology. Transcripts were abductively analysed, with a sample analysed by two researchers, using the Theoretical Domains Framework, which allows identification of individual and contextual factors that influence behaviour, and discussed in team meetings to develop themes.

**Setting:**

Interviews took place either in person or online via Zoom, were audio-recorded and transcribed verbatim.

**Participant:**

A convenience sample of GPs working in Australian primary care.

**Result:**

14 GPs were interviewed for an average of 59 min. Four themes were developed representing the latent mechanisms underlying GPs’ prescription and use of NDIs. (1) Obtaining and sharing knowledge: GPs’ learning about NDIs is limited through medical school and continuing education, highlighting gaps in tertiary and specialty training. Sharing knowledge occurs bidirectionally. GPs share their learnt knowledge about NDIs with patients, who in turn share their lived experience knowledge. (2) Considering the patient: patient characteristics, circumstances and actual or perceived expectations influenced GPs’ NDI prescription. Influences included financial status, therapeutic relationship, patient motivation, presenting condition and medication expectation. (3) Influence of primary care environment: time constraints, billing and policies influenced when and how GPs used and prescribed NDIs. Interprofessional collaboration and distributing patient resources were strategies used by GPs to overcome barriers. (4) NDIs part of GPs’ role and identity: NDIs were prescribed as first-line treatments, preventative strategies or as an adjuvant to medication for both acute or chronic conditions, highlighting NDIs as core to GPs’ role and care.

**Conclusion:**

This study reveals the interplay of factors and mechanisms influencing Australian GPs’ use of NDIs, including systemic, educational and interpersonal dynamics. To optimise the integration of NDIs in primary care, prioritised training, clearer guidance and better access to evidence-based resources are required.

**WHAT IS ALREADY KNOWN ON THIS TOPIC**

- Despite strong evidence supporting their effectiveness, safety and cost–benefit, non-drug interventions (NDIs) remain underused in primary care, with existing research largely focused on patient perspectives and limited exploration of general practitioners’ (GPs) views.

**WHAT THIS STUDY ADDS**

- GPs have overall high levels of knowledge and understanding of the importance of NDIs in acute and chronic conditions management and prevention. However, they described many barriers, including patient factors impacting the implementation of NDIs, time restrictions and resource availability. Additionally, GPs noted that NDI content in undergraduate and specialty training was limited, and education and training on NDIs were often self-initiated or self-taught.

**HOW THIS STUDY MIGHT AFFECT RESEARCH, PRACTICE OR POLICY**

- Providing education and raising awareness of available, effective NDIs for GPs is essential at all levels of training, including medical school and specialisation. Addressing other barriers identified will also enhance the uptake of NDIs.

## Introduction

 Non-drug interventions (NDIs; also called non-pharmacological interventions), which include nutrition, physical activity, digital tools, such as mobile apps, procedures and cognitive-behavioural therapies, represent a cornerstone of primary care.^1 2^ With substantial evidence supporting their effectiveness,^3^,^7^ a lower cost and side-effect profile compared with pharmacological treatments,^8^ and existing integration in both national and international clinical guidelines,^9^,^11^ NDIs serve as powerful first-line options for managing a wide range of acute and chronic conditions. In Australia, primary care settings encompass general practice, community health clinics, including Aboriginal and Torres Strait Islander Health clinics.^12^ As the first point of contact for most patients,^12^ general practitioners (GPs) are the key health professionals responsible for integrating NDIs into patient care. This role is supported by resources like the Royal Australian College of General Practitioners’ (RACGP) Handbook of Non-Drug Interventions (HANDI).^2^

Despite the established health, economic and risk benefits of NDIs and the availability of clinical resources, a significant gap persists between their potential and their routine implementation in practice. A recent survey of Australian GPs found that even among clinicians with a pre-existing interest in the topic, only one-third (34%) regularly recommend NDIs where appropriate, suggesting the true rate of use across the profession is even lower.^13^ This evidence-to-practice gap points to numerous obstacles that prevent clinicians from consistently prescribing NDIs. While some research has explored these challenges, the literature is overwhelmingly dominated by the patient experience. A recent overview of reviews, for instance, found that 92% of the literature focused on barriers and enablers to NDI use from the patient’s perspective.^1^ While the recent survey hints at some obstacles for GPs, including access to resources, support to use in practice and training,^13^ the details and mechanisms of these barriers remain underexplored. This leaves a critical gap in understanding the experiences of GPs, who are at the forefront of prescribing and educating patients about these interventions.

Given the evidence-to-practice gap of NDI use and the under-representation of clinicians’ voices in existing research, further exploration from the GP perspective is clearly warranted. Therefore, this qualitative study aims to explore Australian GPs’ experiences and perspectives on the use and prescription of NDIs. Using a realist approach centring the GP perspective,^14^ we seek to understand the latent mechanisms influencing their clinical practice to inform future interventions designed to improve NDI uptake.

## Methods

### Design

This qualitative study was guided by aspects of Pawson’s realist approach, which seeks to understand how and why interventions produce outcomes in specific contexts.^14^ We explored the underlying mechanisms that influence GPs’ prescribing and use of NDIs in the primary care context through obtaining their experiences of how they prescribe and use NDIs in their practice, and their perspectives on how they interpret and articulate their use of NDIs.^15^ The analysis focused on identifying and interpreting the contextual factors and mechanisms that shape prescribing behaviours, with findings presented as themes. This approach also enables us to recognise that the intended outcomes of NDI prescriptions are context-dependent and may vary between GP participants.^14^

### Sampling and recruitment

GPs were eligible to participate if they were either GP registrars, currently undertaking their specialist training, or had their Fellowship of the RACGP. GPs had to be currently practising and seeing patients in Australia, in any workload capacity. A convenience sample was used, where GPs who responded to study invitations and were eligible participated, as it can be challenging to recruit GPs, due to their busy workloads and barriers in reaching them.^16^ Realist methods focus on data saturation and completeness, rather than the number of interviews.^17^ We aimed for a sample of 10–15 GPs interviewed for 30–45 min as this was considered methodologically sound and feasible, allowing for sufficient thematic depth and data saturation while accounting for recruitment challenges and resource constraints.

GPs were recruited from several sources: GP education workshops at the university where this research was conducted, practice-based research networks, including GoldNet Research & Education Network,^18^ Primary Health Networks,^19^ the RACGP GP Research Project Noticeboard,^20^ email invitations to medical centres and the authors’ professional networks. Some participants were previously known to the research team, as recruitment was done through the university, where some GPs have teaching roles, and others were known through the RACGP HANDI project team.^21^ GPs were given a participant information statement and provided written consent via email and verbal consent prior to the commencement of the interview recording. GPs also received a $A50 e-gift card as a thank you for their participation.

### Data collection

GPs were invited to participate in individual semistructured interviews, either in person at Bond University or via Zoom videoconference. The first seven interviews were conducted by a research assistant with training in qualitative interviews, and the second seven were conducted by AD, a postdoctoral researcher and dietitian, who has extensive experience with qualitative data collection methods. Interviews were recorded and transcribed verbatim using transcription software, Otter AI,^22^ with any identifiable information anonymised during transcription checking by AD. Memos were also kept by interviewers. The interview guide was developed using the Theoretical Domains Framework (TDF).^23^ Interview questions focused on GPs’ experiences of use of NDIs and current NDI prescribing practices, defining NDIs, GPs’ confidence in prescribing, factors influencing prescription, motivators and challenges to NDI prescription. The interview guide is available in online supplemental file 1.

### Data analysis

Interview transcripts underwent analysis guided by the principles of Pawson’s realist evaluation.^14^ An abductive approach to coding and categorising was used for analysis,^24^ where inductive coding was conducted on transcripts using NVivo,^25^ and codes were deductively categorised into the TDF domains and concepts. The TDF was used as it enables categorisation of the individual cognitive and affective, and social and environmental factors that influence behaviour.^23^ It provides a lens to identify relationships within and between codes, categories and themes developed. Inductive coding was led by AD, with IM independently coding a randomly selected sample of five transcripts for triangulation. AD and IM met regularly during data collection and analysis for reflexivity, including sharing potential assumptions and personal interpretations,^26^ and to discuss transcript codes to ensure consistency in analysis. The broader team met during the later analysis stages for discussions and reflections on the project.^26^ Categories were then thematically analysed, whereby codes that were overlapping between TDF domains and concepts were collapsed into themes describing the latent mechanisms of GPs’ experiences and perspectives of NDIs. Theme development and naming were done in research team meetings, considering the results of the previous studies.^1 13^

### Patient and public involvement statement

Development of the study protocol was done with a GP academic, who did not participate in the study, including the creation and piloting of the interview guide.

## Results

14 GPs (50% female) were interviewed between February 2023 and February 2025. Participant characteristics are provided in table 1. Interviews were conducted for an average of ∼59 min (29–85 min). GPs represented a range of years of experience, from registrars with 1.5 years to GP fellows with 43 years’ experience in primary care. All GPs were currently practising in either metro cities, regional or large rural areas. However, most had previous experience working in rural and remote communities.

**Table 1:** Characteristics of GPs

GP	Sex	No of years practised as GP	Current FTE	Practice location (MM)*	No of patients per day	Special interests
1	F	27	1.0	Northern NSW (2)	<26	Mental health, sports medicine
2	F	9	1.0	Gold Coast (1)	30	All general practice areas
3	F	7	1.0	Gold Coast (1)	35	Chronic disease management—obesity and diabetes
4	M	7	1.0	Sydney (1)	10	Maternal health
5	F	40	0.1	Gold Coast (1)	10–12 per half day	Women’s health, all general practice areas
6	F	13	0.5	Canberra (1)	25	Drug, alcohol and severe mental health, First Nations Health
7	M	43	0.4	Canberra (1)	~12	Mental health, drug and alcohol, First Nations Health
8	F	1.5 years Registrar	0.5	Gold Coast (1)	20–30	Chronic disease management
9	M	38	1.0—half AHS and half in private practice	Lennox Head (3)	12 in AHS 20 in private practice	First Nations Health, Non-communicable disease
10	M	33	1.0	Newcastle (1)	24	All general practice areas, medical decision making, environmental health
11	F	38	0.2 FTE	Hobart (2)	30	Mental health, aged care
12	M	1.5 years Registrar	0.5 FTE	Gold Coast (1)	24	Chronic disease management
13	M	30	0.2	Sydney (1)	5–10	Mental health, older persons
14	M	12	1.0 FTE	Tamworth (3)	18–20	Musculoskeletal conditions

* MM: Modified Monash Model: https://www.health.gov.au/topics/rural-health-workforce/classifications/mmm.

ASH, Aboriginal health service; FTE, full-time equivalent; GP, general practitioner; NSW, New South Wales.

### Key themes

**Figure 1:**
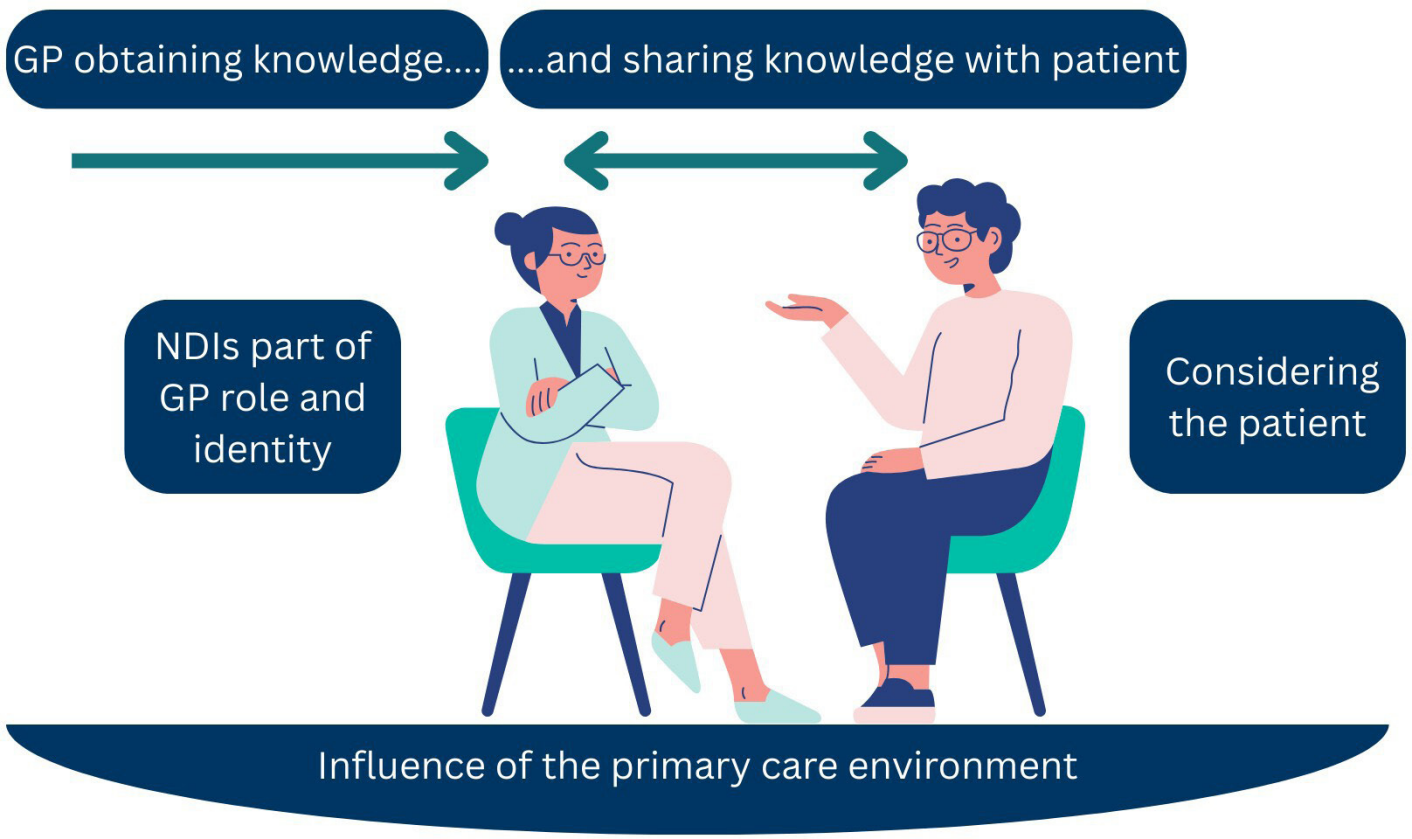
Themes representing Australian GPs’ perspectives of prescription and use of non-drug interventions in primary care. GP, general practitioner; NDIs, non-drug interventions.

Four themes were developed: *obtaining and sharing knowledge, considering the patient, influence of primary care environment and NDIs part of GPs’ role and identity*. The four themes and the connections between them are illustrated in figure 1. This includes the use of arrows in the ‘obtaining and sharing knowledge’ theme to show the reciprocal nature of knowledge sharing between the GP and patient. Additionally, the third theme of ‘influence of primary care environment’ sits underneath the other themes as the practice environment heavily influences the GPs’ role, the knowledge they share and how they consider the patient’s circumstances. Themes are supported by GP participant quotes; additional quotes for each theme are provided in online supplemental file 2.

#### Theme 1: obtaining and sharing knowledge

GPs expressed a high level of knowledge and understanding of NDIs and their use of NDIs in their practice. GPs described various ways they obtained knowledge about NDIs. This included university medical education, specialist training and education, and continual professional development. However, the presence of NDI content in education was inconsistent or tokenistic. GPs who had been practising for longer described that their university training often lacked NDIs as the focus was on other aspects of medicine, including pharmacology and pathophysiology. Similarly, GP registrars undergoing generalist specialisation also experienced NDI education being less emphasised compared with other key practice areas:

*…* at a registrar level, a lot of the focus on our teaching and… trying to frantically study for exams… sometimes (NDI education is) there, but the bigger emphasis is on… diagnostic and pharmacological. Which, you know, fair enough. We need to know that, but yeah, I think the sort of conservative things non-drug interventions, lifestyle, things are just sprinkled as an afterthought sometimes (GP8, Registrar)

GPs’ definition of NDIs aligned well with the study’s definition in many areas, including NDIs that have strong evidence to demonstrate their effectiveness. However, when GPs provided case examples, they also discussed NDIs as lifestyle advice, for example, smoking, nutrition, alcohol and physical activity. Although GPs explained the importance of the information they provided to patients was evidence-based, they also acknowledged that patients may use some interventions that have insufficient empirical evidence. GPs considered it was important to remain open to include what they perceived to be ‘non-evidence-based’ NDIs and practitioners, such as naturopathy, when requested by the patient.

The sharing of NDI knowledge from GP to patient was described by participants as vital in NDI implementation. This sharing of knowledge is represented in figure 1 with the double-ended arrow, where GPs passed on their knowledge of NDIs to patients, and also received input and information from patients as feedback to this NDI education. Knowledge sharing was often done in small, simple steps to ensure patient understanding and the ability to implement changes. This knowledge sharing was dependent on many factors, including patient stage of change, reading and health literacy levels, and the GPs’ perception of patient expectations. Other factors, including consultation time constraints and availability of NDI resources, also influenced the knowledge sharing process. These factors are further described in the sequential themes.

So, it’s a slow, incremental, repetitive, you know, every time you see them, you know, ‘what did you do? Okay. Why didn't it work? How can we make it work better?’ sort of process (GP7)

#### Theme 2: considering the patient

GPs were heavily influenced by the patient, their condition(s) and their circumstances when it came to NDI prescription. Patient circumstances that GPs considered included cultural background, English proficiency, financial status and social influences. GPs made clinical decisions regarding selecting NDIs that suited the patient’s condition, for example, with Type II diabetes, GPs often discussed both dietary and exercise-based interventions. However, GPs prescribed and implemented NDIs for both acute and chronic conditions that patients presented with.

GPs’ decision to discuss NDIs was influenced by their perception of what patients expected from the consultation. This depended on previous interactions with the patient and the therapeutic relationship. If the GP had a prior positive interaction with the patient, they were more likely to discuss NDIs. Opposingly, following a prior negative interaction, GPs often decided not to discuss NDIs with patients, believing it may be a waste of time and energy due to anticipated potential patient rejection. If GPs perceived that the patient would not be open to hearing about NDIs or that implementing the NDI would be too challenging for the patient, they would often leave NDIs out of that consultation:

(a patient) might say, ‘Oh, I work too long… I have to take the kids to school, I can’t go for a walk, I can't, it’s just too difficult.’ So, you don’t waste your time… it’s like motivational interviewing…you ask and explore before you go any further. And if you get these roadblocks coming up, and clearly a lack of interest or motivation or living in a different world to me where they don’t have the chance or the opportunity… some people are trapped in their circumstance though. And you have to be empathic and understanding and roll with what they can and can’t do (GP5)

GPs emphasised the importance of evidence-based NDIs; however, in some circumstances, they were open to patients using ‘non-evidence-based’ NDIs. This was often guided by the patient’s request, or because the patient had already been implementing this kind of intervention or lifestyle change before consulting the GP. To ensure that GPs felt they were being patient-centred, they often encouraged the patient to continue engaging with practitioners and the ‘non-evidence-based’ NDI if they were seeing a benefit:

I think it’s really important that everyone feels included because like as a GP, you're kind of like the bridging of everyone; trying to bring them all together… some people are really matched with their naturopath and you've got to bring them together. Otherwise, they're going to be feeling really outside, and people are like ‘Oh, no’. No, no, everybody is included here (GP2)

GPs also described balancing patient expectations for medication prescriptions, as they believed that if a patient left a consultation without this, they did not feel heard in their presenting condition. GPs reassured their patients that prescribing a NDI meant that they were listening to the patient and they believed the patient. This management of patient expectations was challenging in some cases, as it required a significant amount of consultation time to educate patients on why a medication prescription may be unhelpful or even harmful. A key example of this was antibiotic prescription for viral infections:

For example, I had a patient who said that it’s now 2 weeks, since I’m having this cough, a bit of shortness of breath, sore throat is becoming better, but it hasn’t fully gone. ‘Do you think I need antibiotics?’ So, she didn’t have any fever. But things are getting better. And I told her, you know, just do some saltwater gargling and good hydration, and see how she goes through the next few days. And if she thinks it’s not getting better in the next week, come back. And I also told her about red flags, if it gets worse what to do (GP3)

#### Theme 3: influence of primary care environment

GPs described that factors influencing NDI prescription extended beyond the patient–GP interaction. This strong influence of the primary care environment is reflected in figure 1 as it sits underneath the other themes and subthemes. These broader influences included primary care policies, organisational structures, and patient and clinician resources accessible to the GP. Primary care policies, such as the Medicare funding and billing policies, influenced the amount of time that GPs could spend with patients. GPs expressed that the standard 15-minute consultation was insufficient to adequately transfer knowledge about NDIs to patients. Additionally, clinic environments are set up where GPs are often not interacting with their GP colleagues and thus do not know how they approach NDI use and prescription:

You asked me that question: ‘What are other GPs doing?’ And I’m like I have no idea I’m not really with them. But do you know what I mean, we don’t really have contact (GP2)

To overcome this time barrier, GPs would use other team members, including practice nurses and referrals to allied health professionals where appropriate. Additionally, GPs would use available NDI resources and handouts to provide to patients to take home to implement the NDI. There was no consensus between GPs on which resources they used; many described using Better Health Channel, Murtagh’s and the RACGP HANDI. They also expressed the need for more accessible, user-friendly and patient-appropriate resources. For example, short, concise, typically 1–2 pages of information, they can electronically send or hand to the patient in the consultation, was desired by GPs. They expressed a high need for their information and resources, including patient resources, to be part of a practice software-integrated programme:

*…*we don’t need to be sort of getting more information in a way that’s going to complicate it even more. You know what I mean? We want to try and integrate (resources) and coordinate it. That’s why I think it’d be really good if it was on (practice software name), and the stuff that comes out actually talks to (practice software name) (GP14)

#### Theme 4: NDIs part of GPs’ role and identity

GPs described the use of NDIs in their practice as part of their GP role as an evidence-based healthcare professional. GPs described that they have built and adapted their practice to focus on NDIs and to implement them as part of usual care for all of their patients, where clinically relevant. NDIs were described as first-line for many preventative measures and for the management of acute and chronic conditions. However, for certain conditions and diseases, NDIs were discussed in combination with medications. For example, in severe mental health cases, the GP would prescribe the indicated medications and also talk about the importance of exercise and diet to support the patient’s mental well-being. NDIs were also seen as a method for reducing initial medication prescribing or deprescribing, with a focus on patients who experience medication side effects or who have polypharmacy. GPs described the key lifestyle areas they would ask about in nearly every consultation:

All of my consults…I speak to them, I ask them about diet, exercise, smoking, alcohol, preventive medicine… for nearly all…all my consults have non-drug intervention (GP3)

GPs were driven by the internalised feelings of responsibility to their patients to meet a standard of care. GPs described feeling expected to uphold this standard of care both from their patients and their colleagues. GPs also expressed wanting to do more advocacy and teaching of NDIs, in particular, the more experienced GPs, to support GP registrars and medical students in using NDIs in practice.

I was thinking about what we might be talking about this evening. And, and I kind of had this bit of a guilty feeling, because I’m thinking, you know, even though I’ve been associated with HANDI for so long, there’s probably topics there that I’ve forgotten about, topics we’ve done, that I’ve forgotten about. And I don’t promote where I could be (GP9)

One GP expressed a critical view on NDIs and the GP profession that displays an intersection between their own personal views and behaviours and their role as a GP. They described that GPs were a role model to patients in their health behaviours and that they too should be following their own NDI advice. This role modelling behaviour was a key influence for GPs to prescribe NDIs. They believed that GPs who were not comfortable looking inward at their own health behaviours were less likely to prescribe NDIs in practice:

I think it’s difficult to encourage people with respect to…smoking interventions, alcohol interventions, exercise interventions, and… healthy approaches to eating when you don’t follow it yourself… I think that would be in the…majority of practitioners would have something that they…would have to confront themselves, to actually prescribe it (GP1)

## Discussion

This qualitative study aimed to explore the perspectives and experiences of Australian GPs on their use and prescription of NDIs in practice. GPs expressed a range of latent mechanisms that influenced their decisions around NDI prescription and use. These mechanisms were portrayed in the four themes: obtaining and sharing knowledge, considering the patient, influence of primary care environment and NDIs as part of GPs’ role and identity.

GPs in this study described various ways they acquired knowledge about NDIs, often motivated by personal interest due to gaps in their formal training. Previous studies involving Australian medical students have highlighted that medical school curricula do not adequately teach NDIs, particularly in the areas of nutrition,^27^ physical activity^28^ and pain management.^29^ This issue is not unique to Australia, with a 2023 US study mirroring these experiences despite a recognition of the importance of NDIs.^30^ Reflections from GPs in this study suggest that this goes beyond medical school curricula, with specialty training also offering limited education on NDIs. These findings highlight two key opportunities to enhance NDI training for doctors: during university medical education and in postgraduate specialty training.

Although major professional organisations such as the World Organization of Family Doctors (WONCA) and the RACGP advocate for training in NDIs, current efforts remain patchy and inconsistent. WONCA has emphasised the importance of comprehensive education and training in medical curricula to support the management of common mental health conditions in primary care.^31^ In Australia, GP specialty training through the RACGP includes some coverage of NDIs—primarily in the context of neurological conditions, pain management and, to a lesser extent, musculoskeletal and cardiovascular diseases.^32^ This highlights a global need for the systematic and comprehensive inclusion of effective, evidence-based NDIs in medical education and training programmes.

GPs tended to conflate NDIs with general preventative lifestyle advice such as SNAP (smoking, nutrition, alcohol and physical activity), rather than recognising the broader scope of NDIs beyond lifestyle factors. This misconception that NDIs and lifestyle advice are synonymous can lead to ineffective prescription of NDIs and barriers to patient and health system implementation. NDIs are intended to be specific, evidence-based interventions that are prescribed to the patient to manage or treat a specific health condition. A previous study from the UK demonstrated that providing patients with broad, general exercise advice in a consultation did not result in positive, long-lasting changes.^33^ It has been estimated that primary care providers need approximately 14 hours a day to conduct guideline-recommended preventative activities alone.^34^ Although this estimate includes pharmacological interventions such as immunisation and secondary prevention, offering broad, non-specific lifestyle advice for primary prevention places additional strain on an already overstretched primary care system and may not enhance patient satisfaction.^35^ Awareness of the distinction between NDIs and lifestyle advice is crucial to the implementation of NDIs in primary care.

The decision to prescribe or use NDIs is shaped by both GPs’ clinical decision-making and patient receptiveness, preferences and values in the broader context of their lives, which is in line with shared decision-making principles.^36^ Interestingly, the GP’s perception of the patient’s receptiveness to or capability of using NDIs also informs whether they are prescribed. This concept is well-established in both clinical practice and the literature, reflecting the emphasis on patient-centred care in Australian health professional training.^37 38^ Many of the patient-related factors that influence a GP’s decision to prescribe or discuss NDIs were previously outlined in an overview of reviews on barriers and enablers in primary care.^1^ These factors include the actual or perceived cost of the intervention, limited access to necessary goods or services (eg, exercise equipment), and language or cultural barriers. As demonstrated by the GPs in our study, GPs should aim to strengthen patient enablers and support patients in identifying and overcoming these barriers.

The primary healthcare environment has been shown to shape and influence the clinical practice of GPs and can differ from practice to practice, and across countries with primary healthcare systems.^39 40^ GPs in this study outlined how they overcame the barriers that current primary care policies and funding schemes create, including interdisciplinary collaboration with practice nurses (PNs) and allied health professionals to provide more detailed and discipline-specific NDIs. The role of the PN in Australian primary care has grown considerably since funding for their position in general practices was introduced in the early 2000s.^41^ This includes many primary care models being led by PNs or becoming team-led with GPs and PNs working closely together to provide comprehensive care to patients.^42^ The addition of allied health to provide tailored NDIs is enabled by GP Chronic Condition Management Plans, formally known as GP Management Plans and Team Care Arrangements.^43^ These plans are intended to provide comprehensive care for people with chronic conditions by enabling regular, 6-monthly overviews of care by their GP and an opportunity for subsidised referrals to allied health. However, access to this is still low, with many patients either not meeting the eligibility criteria, struggling with out-of-pocket expenses and underutilisation of plans.^44 45^

Resources used by GPs included other care team members, such as nurses and allied health, patient handouts, practice software, and other electronic programmes such as HANDI,^2^ the Better Health Channel website^46^ and John Murtagh’s Patient Education resources.^47^ A sole platform for GPs to use for their own resources to learn about new NDIs and a repository of patient resources would ensure consistency between clinicians. Additionally, a trustworthy and reputable source of information could provide up-to-date information and evidence-based NDIs. Building on the current RACGP HANDI, the sequential project of this qualitative study will explore codesigned adaptations of the resource to explore GP acceptability and feasibility of an online tool, titled the electronic HANDI (e-HANDI).^2^

The strengths of this study include building on results from two previous studies, including the overview of reviews of NDI perspectives from broad perspectives, including patients and clinicians, and from a survey of Australian GPs.^1 13^ Together with the current study, these studies will inform a programme theory, drawing on multisource evidence to build a comprehensive picture of the mechanisms behind NDI prescription and use in the primary care context.^14^ Another strength is the in-depth interviews with GPs from a broad range of experience levels. However, it is likely that those who responded to be interviewed have an interest in NDIs and use them regularly in their practice, including the GPs from the RACGP HANDI committee. This is common in qualitative studies where participants who have a vested interest in the topic are more likely to respond to recruitment.^48^ A potential limitation could be the small thank-you $A50 gift card that may have limited the incentive for GPs to participate. Additionally, due to the convenience sampling, current practice settings and approaches are missing from GPs in smaller rural towns and remote areas of Australia. There are some previous qualitative studies that explore rural and remote GP views on NDIs in certain conditions, including heart failure^49^ and mental health.^50^

Future research should aim to explore the gap in rural and remote GP views of NDIs more broadly. Additionally, GPs highlighted that they were largely limited in their NDI prescription based on the patient’s barriers and condition/s. It is therefore important to explore patients’ views on their use and prescription of NDIs in their own health and well-being. This will ensure equitable and context-sensitive integration of NDIs into everyday practice. Further research looking at reviewing and codesigning an updated HANDI resource for GPs is already underway, based on feedback from GPs involved earlier in the study.

In conclusion, this study provides valuable insights into the complex factors influencing Australian GPs’ use and prescription of NDIs in primary care. GPs described a range of latent mechanisms that shape their clinical decisions, including knowledge acquisition, patient receptiveness, systemic constraints and professional identity. The findings highlight critical gaps in medical education and training, particularly around the scope and specificity of NDIs, and underscore the need for clearer distinctions between NDIs and general lifestyle advice. Enhancing access to trustworthy, evidence-based resources and fostering interdisciplinary collaboration may support more consistent and effective NDI implementation.

## Supplementary material

10.1136/fmch-2025-003741 online supplemental file 1

10.1136/fmch-2025-003741 online supplemental file 2

## Data Availability

No data are available.
