# Supplementary material for: Australian General Practitioners’ perspectives, experiences and use of non-drug interventions in primary care: a qualitative study
Source: Fam Med Community Health. 2026 Jan 6;14(1):e003741. doi: 10.1136/fmch-2025-003741 (PMC12778305; doi:10.1136/fmch-2025-003741)
Supplement: online supplemental file 1 [file fmch-14-1-s001.docx]

**Supplement 1:** Interview guide for General Practitioners perspectives, experiences, and use of non-drug interventions in primary care: A qualitative study.

**Interview Guide: Barriers and enablers to GP use of NDI and HANDI**

**Demographic Questions**

Thank you. Before we start our discussion, I’ll go through some demographic questions with you.

1. What is your **gender**?
2. How many **years** have you worked in general practice?
3. Is your primary practice in a **rural or metropolitan area**?
4. Approximately **how many patients do you consult** in your practice per day?
5. Do you or the practice at which you work have **any special interests**?
   1. E.g. Women’s health, mental health etc.

**Barriers and enablers of NDI**

Let’s talk about your use of non-drug interventions. Please remember that there are **no right or wrong answers**, we just want to hear about your thoughts and experiences as a general practitioner.

1. What does the **term “non-drug interventions” mean** to you?

*PROBES:* What about **exercise**, do you consider this to be an NDI?

- - 1. *Exercise*
    2. *Physical therapy*
    3. *Diet*
    4. *Psychological interventions e.g. CBT*
    5. *Herbal medicines*
    6. *Acupuncture*
    7. *Massage therapy*
    8. *Social prescribing*
    9. *Other alternative medications*

1. With regards to non-drug interventions, when do you think they are **best used** and why do you think they may be a **useful approach** in patient management?

From now on, when I say non-drug interventions, I’m talking about effective, evidence-based interventions that use exercise, physical therapies, diets, or psychological interventions, rather than pharmacological/drug treatments. This does not include interventions that are not evidence based, such as homeopathy.

Does that definition make sense to you?

From now on, I will talk about prescribing non-drug interventions. Please keep in mind, that when I use the term ‘prescribing’ I am talking about any discussion, recommendation, or prescription you give your patients to use a non-drug intervention.

Does that definition make sense?

Okay great. Feel free to ask me any questions throughout the interview, if you need anything clarified.

1. Do you **prescribe non-drug interventions** in your practice?

***If yes*** **->** Which NDIs do you use? How often would you say you prescribe an NDI?

***If no* ->** I just want to confirm that I have understood correctly, and you never discuss, recommend, or prescribe things like exercise, physical therapies, dietary changes, or psychological interventions in your practice?

***If yes* ->** back to **a.**

***If no* ->**  Can you please let me know why you do not prescribe NDIs in your practice? **-> PART B**

1. How **confident** do you feel prescribing non-drug interventions to your patients?

*PROBES:*

Is there anything that has **effected your confidence** to prescribe NDIs*?*

Is there anything you can think of that would **increase your confidence** about prescribing non-drug interventions?

1. What **factors influence whether** you prescribe non-drug intervention with a patient?

*PROBES:*

Can you think of anything that would **make it easier** for you to prescribe non-drug interventions in patient management?

What do you think are the **advantages and/or disadvantages** about using non-drug interventions?

1. How do you go about **having discussions** about non-drug interventions within patient consultations?

*PROBES:*

Is there **anything you do or say** when prescribing a non-drug intervention?

1. What **challenges** have you encountered in being able to prescribe non-drug interventions to your patients?
2. Is there anything that **motivates or discourages** you from including NDIs in your practice?

*PROBES:*

What are your **personal or professional goals** about including non-drug interventions in your practice?

1. Is there anything you have done or thought of doing to help you **get into the habit** of including NDIs in your practice?
2. Can you tell me a bit about whether you think it is **part of your professional role** as a GP to include NDI in patient care?
3. How do you feel about non-drug interventions being a **part of standard patient care**?
4. How do you think **patients perceive being prescribed** non-drug interventions compared to drug interventions?
5. Is there **anything else you want to tell me** about prescribing non-drug intervention in your practice?
