# Supplementary material for: Australian General Practitioners’ perspectives, experiences and use of non-drug interventions in primary care: a qualitative study
Source: Fam Med Community Health. 2026 Jan 6;14(1):e003741. doi: 10.1136/fmch-2025-003741 (PMC12778305; doi:10.1136/fmch-2025-003741)
Supplement: online supplemental file 2 [file fmch-14-1-s002.docx]

Supplementary 2: Illustrative Quotes from GPs Supporting Key Themes on NDI Use and Prescription Practices

| Theme | Quotes |
| --- | --- |
| 1 - Obtaining and Transferring Knowledge | *“For me, massage therapy and acupuncture, I have anecdotal experience, I guess, of those modalities and the benefits that they give, I would probably put the herbal treatments, naturopathic treatments in, in drug in the drug remedies, category, not the non-drug categories.”* – GP1  *“If I don't know the answers, you're a generalist, so you try to do the best that you can, and then you try and find out that information.”* -GP2  *“I did medicine in India and I specialized in pharmacology. So, I did spend a lot of time, actually three years, learning about all medications in detail. So, I know all the medications have side effects, even paracetamol, so I, I myself, I'm really hesitant to prescribe medications other than... I prescribe medications only if it is really essential.”* - GP3  *“Well, in my opinion, it's not part of, you know, my professional studies. It's this sense comes with, you know, experiences. So just by experience, I have tested it, and it has worked. I'm not being taught non-drug interventions in school. I'm not being taught at all.”* -GP4  *“I try and be really detailed in my advice and explain all the different steps and things they can do.”* - GP6  *“There is this education component where you say… well, the evidence shows that if you actually go out walking, it's going to make you feel better, it won't make you instantly feel better, like on your first walk. But if you do it regularly, you'll find you'll be less depressed, you'll feel more active, you'll get fitter, you'll feel more motivated. So, and you don't quite state as you just say, "the evidence shows" like on adverts on TV [laughs].”* – GP7  *“We [General Practice team] have a meeting every Friday morning as sort of a clinical get together for a kind of educational meeting.”* – GP10  *“As a GP, I can talk about it [NDIs]. But it's, it's the ability to show the person and give them a piece of paper or whatever something to engage them so that you can get that motivational interviewing to get a management plan in place.”* – GP11  *“I think it's poorly taught in general, probably in med school, and also in most training pathways. But the two or three years, or whatever it is that you're in hospital, depending on what specialty you want to go down, it's almost never mentioned at all.”-*GP12  *“Interestingly, for GP information, GP education. Of course, there's also new organizations turning up all the time. So, you know, the College [RACGP] of course has been a place where you could find information for a long time. But you know, now there's health ed and webcast, and on it goes. There's new ones coming out all the time.”* – GP13  *“I worked as a physio for 15 years before I learned medicine. So, looking at conservative strategy is something that I think is really important in medicine. And I think a lot of doctors don't do it very well. So, I think, you know, we're trained to come in at sort of, you know, conservative, medical, surgical. And I think most doctors come in at medical and go to surgical pretty quick, whereas I tend to come back to conservative.”* – GP14 |
| 2 - Considering the patient | *“I think that the appropriateness of [NDI prescription] is dictated by the clinical concern. And I would imagine, in the vast majority of health and illness consultations, lifestyle interventions play a part in just about everything. So I don't think there'd be ever a case that I wouldn't, how I go about it often, and again, are probably going to reflect on probably ment- mental health patients for a start… I generally talk to them about what their day looks like, which is generally not one that's going to support good health. And I would make those interventions quite early. And probably the first one that I often make is sleep. For other, I guess, more traditional physical conditions, so hypertension, diabetes… it's going to be a part straight out.”* – GP1  *“…the thing is not to be judgmental…let's try this, and then see what happens until we do this, then we set like a plan…So, I think it's just being that…they've got a place, a safe place to talk about their thoughts, and that you go, that's fine, you know, and you work out a way…”* - GP2  *“In my practice, I actually find patients, most of them don't want to take medications, initially, so they, they like non-drug interventions.”* - GP3  *“Okay, some patients, you know, readily accept the prescription, and some at the end of the day wouldn't, you know, take it, they wouldn't admit, they wouldn't want to do it.”* – GP4  *“I would sound out their feelings first, because physio has a cost, they may or they may not be eligible for a management plan, with team care arrangements to give them some Medicare cover. They may not have private health cover, they may have the money to pay for physio. They may not want to go there and do that. So, it is a conversation before it's a recommendation. So, it's part of the shared understanding, and therapeutic alliance between doctor and patient.”* - GP5  “Sometimes if we say, oh, you know, you don't need antibiotics, whatever people think you don't think they're taking them seriously. But if we're not using medications, maybe people think we don't kind of believe their symptoms or believe what's going on for them. But it's really important in when using non-drug interventions, to let them know you do believe them, and you know, what they're experiencing. And these things are really effective for helping them feel better.” -GP6  *“So, it's a slow, incremental, repetitive, you know, every time you see them, you know, "what did you do? Okay. Why didn't it work? How can we make it work better?" sort of process. But yeah, so I think, you know, people accept the fact that I'm doing non-medical, you know, lifestyles, prescribing, they, they obviously get, it comes back to motivating people.”* – GP7  *“It [NDI prescription] really depends on if patients want to talk about that or hear about that. I can get like a bit of an eye rolling response, at times. So, that throws me off. So, to support it for like future attempts, I guess. Sort of having the previous kickbacks, I guess…Because, yeah, it's hard to not use past experiences, like, Oh, if I bring this up, they're probably just gonna roll their eyes again, like, you know, is it worth it? Which is you know, not a great thing, but just happens.”* – GP8  “*I guess you can sometimes tell from the way a person responds, you know, whether they're more keen on something they can do for themselves or something that they can take. It's a negotiation, you sort of offer them what the treatment options are and pick up from there mostly nonverbal cues about which ones they're interested in.* – GP10  *“I have to think about their, [the patient’s] IT literacy and their literacy levels, to think about, you know, how much I'm going to recommend a website. And then, in terms of getting into the nitty gritty about the evidence base, and why we've chosen it… and many people don’t want drugs.”* – GP11  *“Patients are sometimes anti non-drug interventions, they just want something that will work quickly, rather than having to put in sometimes what they perceive as effort to have a reward. And on the flip side, some patients are very much against using medications, they want to try more natural things.”* – GP12  *“I'll do the longer consultations and the consultations where you can chop it up into bits and people are quite comfortable about stuff happening over a period of time, or two or three consultations, that sort of work, and that does tend to lend itself to the sort of people who are happy with that sort of work.”* -GP13 |
| 3 - Influence of primary care environment | *“…[NDI prescription] might range from referrals to allied health professionals with respect to dietary interventions, exercise physiologist for prescription of exercise, and physiotherapist, osteopaths with respect to treat, you know, treatment and maintenance of injury free movement…I certainly don't embark on any specific diet advice, I probably would refer.”* – GP1  *“…we've got to do lots of things, and you can't rush anything, you know. And so, they're really quite quick…I was going from roughly like, 7:15 this morning till now, no break…”* – GP2  *“I have a GP who works with me. Frankly speaking, I don't have, we are really busy during the day. I don't speak to her much about patients. I am trying to, you know, but there are some difficult patients. I was thinking of having face conferences with her. But, haven't got there yet.”* -GP3  *“As a GP, it's actually often easier to refer them to a physio who will take them through it, yeah. Who will give them a few training sessions, I don't have the time to train them. I just don't have the time to take them through it. It takes too long.”* - GP5  *“…you imagine that guideline would be pretty massive. And then the more you put into it, the less useful that sometimes becomes because it's a bit overwhelming, it'd be okay for maybe simple things. But I mean, definitely would be amazing if there was just one place, but I can't imagine how it would be laid out so it would be really helpful and easy to use, especially in a clinical setting. Because in a clinical setting, it needs to be so fast.”* - GP6  *“Working in Aboriginal Health, where there was a large sort of group of practitioners who were all working on sort of evidence-based stuff, we were very early adopters of this when it started to come out. And because the practice was doing it, and we talked about it in practice meetings. And then we actually monitored what we were doing as part of our clinical auditing. It just became sort of normal.”* – GP7  *“In [practice software], like a little patient education button, I have lots of patient handouts that I find very helpful, and often more comprehensive than whatever I say and have time for so let's say I need to give them that they can read that in their own time.”* -GP8  *“There's more use of allied health than it used to be, driven by Medicare funding. But that's if the Allied Health people are implementing good, evidence-based stuff, that's great. That'd be the main system driver, I can think of that would have promoted non-drug interventions.”* – GP9  *“A lot of my young [patients] come in and say they've got a mental health issue, and do expect you to give them medication. And I find that a bit sad. And I think it's, it's to do with the fact that often GPs may not have the time and skill to give them an alternative.”* – GP11  *“Like everything else, you look at your resources and your costs. Time is a cost and a resource. And so, if you're only going to be billing Medicare for a sort of modest amount of money, I'm not saying it's no money, but it's only a modest amount of money.”* – GP13  *“The problem with Medicare is it's not supporting those people like me who really want to spend more time and actually really break down their diet and really break down how they're going to lose their weight or really break down their depression, and you know what they need to do to really help themselves. You can't do that in seven minutes. So, you can see why we've got problems and why we need more time with patients to do this sort of stuff.”* – GP14 |
| 4 - NDIs part of GP role and identity | *“I would say every day, [prescribes NDIs], there's people that I see every day where I, if I'm not, you know, if it's not my first contact with them, then it's follow up. And it's generally going to be, you know, how are you going to sleep hygiene? You know, what, you know, what are you doing with respect to activity?” -* GP1  *“…say someone has osteoporosis, so we know that it's combined, you know, you know, you want them to improve their bone, like two times a weight bearing training, gonna give vitamin D to a certain level and your calcium to a certain level. But you know, then we want to improve their bone health. So then we think of, you know, medications as well on top of that.”* – GP2  *“I fellowed only the year before, so I'm quite fresh. So, for us, we always start with non-drug interventions, and then go into medications.”* - GP3  *“We have to teach this to every patient, though non-drug intervention will not solve all problems, we should understand that non-drug interventions really work.”* - GP4  Interviewer: *“Do you prescribe non-drug interventions to your patients?”* GP6: *“Yes. All the time.”*  *“[NDIs is] not using medication as your first line of approach to managing people's conditions, I put it as fairly first line, most of my effort is trying to get people to move…pretty much everybody I see, there’s some sort of non-drug prescription involved in in the management treatment plan.”* – GP7  *“So, a specific example for me. You know, I started doing weights training three times a week last year. I didn't know how it would go, but I actually quite enjoy it. And now, you know, I've gotten stronger, I feel better, you know, my posture is better. Like, I can tell that this has been a positive thing. So yeah. Yeah, I feel like we always kind of know that, you know, exercise or you know, dietary things are good for us. But then actually experiencing the positive effect of that is also reinforcing that idea.”* – GP8  *“I think it's sort of outside the cultural expectation, the expectation if, say, the doctor diagnoses something, and I'll give you a prescription. That's the standard way of doing it. So, to actually say, ‘Well, no, we don't need to use medication, per se. But this is a way forward. Which will get us there. It might take a little bit longer, but you will have a permanent path that you can revisit.”* – GP11  *“So, I think it's important, very important for us to know as well, what's available, what's actually evidence-based.”* – GP12  *“I have always offered a sort of open-ended style of practice face to face. So, setting aside plenty of time for people to ask for more time if they needed it, and being quite comfortable about that having consultations that went over 20 minutes, half an hour, that sort of thing that’s been a very standard sort of paradigm.”* – GP13  *“I did go straight to just conservative strategies balance. So, in medicine, one of the frameworks that you sort of learn, or what I've got in my head, is when I get, you know, diagnosis or a problem list of medical issues, how do I manage them? Can I manage it conservatively? Can I help manage the patient without medication?”* – GP14 |
